# Atlas of metabolism reveals palmitic acid results in mitochondrial dysfunction and cell apoptosis by inhibiting fatty acid β-oxidation in Sertoli cells

**DOI:** 10.3389/fendo.2022.1021263

**Published:** 2022-09-27

**Authors:** Xiaoqin Xu, Dandan Luo, Qiuhui Xuan, Peng Lu, Chunxiao Yu, Qingbo Guan

**Affiliations:** ^1^ Shandong Provincial Hospital, Shandong University, Jinan, China; ^2^ Shandong Clinical Research Center of Diabetes and Metabolic Diseases, Shandong Provincial Hospital, Jinan, China; ^3^ Shandong Laboratory of Endocrinology and Lipid Metabolism, Shandong Provincial Hospital, Jinan, China; ^4^ Shandong Prevention and Control Engineering Laboratory of Endocrine and Metabolic Diseases, Shandong Provincial Hospital, Jinan, China; ^5^ Shandong Provincial Hospital Affiliated to Shandong First Medical University, Jinan, China

**Keywords:** palmitic acid, Sertoli cell, energy metabolism, metabolomics, lipidomics, apoptosis, mitochondria

## Abstract

In recent years, the impact of lipotoxicity on male fertility has received extensive attention, especially on Sertoli cells (SCs). In SCs, energy metabolism is important as disorders of energy metabolism result in infertility eventually. However, the underlying mechanism of lipotoxicity on energy metabolism in SCs remains unknown. Advances in high-throughput metabolomics and lipidomics measurement platforms provide powerful tools to gain insights into complex biological systems. Here, we aimed to explore the potential molecular mechanisms of palmitic acid (PA) regulating energy metabolism in SCs based on metabolomics and lipidomics. The results showed that glucose metabolism-related metabolites were not significantly changed, which suggested that PA treatment had little effect on glucose metabolism and may not influence the normal energy supply from SCs to germ cells. However, fatty acid β-oxidation was inhibited according to accumulation of medium- and long-chain acylcarnitines in cells. In addition, the pool of amino acids and the levels of most individual amino acids involved in the tricarboxylic acid (TCA) cycle were not changed after PA treatment in SCs. Moreover, PA treatment of SCs significantly altered the lipidome, including significant decreases in cardiolipin and glycolipids as well as remarkable increases in ceramide and lysophospholipids, which indicated that mitochondrial function was affected and apoptosis was triggered. The increased apoptosis rate of SCs was verified by elevated protein expression levels of Cleaved Caspase-3 and Bax as well as decreased Bcl-2 protein expression level. Together, these findings indicated that PA may result in mitochondrial dysfunction and increased apoptosis by inhibiting fatty acid β-oxidation of SCs.

## 1 Introduction

Obesity has various metabolic complications, including a negative impact on fertility, which has received increasing attention (1, 2). Obesity is often accompanied by dysregulation of free fatty acid (FFA) metabolism, including elevated saturated fatty acids and decreased polyunsaturated fatty acids (3). Palmitic acid (PA) is the most common long-chain saturated FFA in human plasma, and it has been reported that dietary intake of PA is positively associated with asthenospermia (4, 5), indicating an association between PA and male infertility. However, the specific mechanism by which PA affects male infertility remains unclear and needs to be further explored.

Sertoli cells (SCs) play an important role in the development of germ cells (GCs) as they provide physical and nutritional support for the GCs to maintain spermatogenesis (6). Lactate is produced by glucose metabolism in SCs, and it serves as a substrate for GCs to generate energy (7). At the same time, SCs produce their own energy through fatty acid β-oxidation (8). In addition to fatty acid β-oxidation, some amino acids also serve as independent substrates to supply energy for SCs through entering the tricarboxylic acid (TCA) cycle (9). Therefore, energy metabolic homeostasis in SCs is vital in male reproduction (6).

It has been reported that PA overload causes intracellular metabolic disorders and interferes with energy metabolism balance in cardiomyocytes, HepG2 cells, and adipocytes (10) (11). Our previous study showed that PA impaired spermatogenesis by regulating metabolism in SCs, and we speculated that PA may impair mitochondrial function in SCs (12). However, the specific mechanism of PA regulating energy metabolism in SCs remains unclear. Moreover, many studies have reported that PA impairs the function of SCs, including mitochondrial dysfunction, oxidative stress and apoptosis, but it is not clear whether PA leads to these damages by altering energy metabolism in SCs (13) (14) (15).

Recent advances in the integration of metabolomics and lipidomics have greatly extended their reach in biomedical and clinical research, and they have been declared as the key to advancing precision medicine (16). Here, we used a combination of metabolomics and lipidomics to explore the potential mechanism of PA regulating the energy metabolism in SCs and to provide new insights into the molecular mechanism of lipotoxicity injury in SCs function.

## 2 Materials and methods

### 2.1 Cell culture and treatment

SCs were isolated from male Wistar rats aged 19 to 21 days (Shandong University Laboratory Animal Center) as previously described (17). After the tunica albuginea was removed from the testes, the testes were cut into pieces (1-2 mm) and washed three times with PBS. The tissue pieces were hydrolyzed with the following three enzymes with gentle stirring at 37°C for 30 min (90 times/min): 0.1% trypsin (Solarbio, China) and 0.02% DNase (Applichem, Germany); 1 mg/ml collagenase (Sigma Aldrich, USA) and 0.02% DNase; 1 mg/ml hyaluronidase (Sigma Aldrich, USA), 1 mg/ml collagenase and 0.02% DNase. Cells were resuspended in Dulbecco’s Modified Eagle Medium/Ham’s F-12 (DMEM/F12 1:1) with 100 U/ml penicillin, 100 mg/ml streptomycin, 25 μg/ml amphotericin B (Solarbio, China), and 10% FBS (Excell, China) in humidified medium at 33°C and 5% CO_2_. After 48 h, cells were treated with 20 mM Tris HCl (pH 7.4) for 1-2 min to remove residual GCs. The SC-specific protein marker, Wilms tumor 1 (Abcam ab89901), was detected by immunofluorescence. After the SCs reached 90-95% confluence, the culture medium was replaced with insulin-transferrin sodium selenite (ITS; pH 7.4, Sigma, i3146), and 0.5 mM PA or bovine serum albumin (BSA) was added. After 24 h, cells and culture media were collected for metabolomics, and lipidomics assays.

### 2.2 Sample preparation

#### 2.2.1 Metabolome extraction

After samples were collected, they were stored in 3 mL of 80% methanol at -80°C. To prepare samples for analysis, they were removed from storage and centrifuged at 14000 g for 20 min at 4°C. The supernatants were transferred into centrifuge tubes and dried in a low-temperature, reduced-pressure centrifuge concentrator, and they were then sealed and stored at a low temperature before analysis. Before LC-MS measurement, 50 μL of 80% methanol was added to each sample to resolubilize the sample, and the sample was centrifuged for 10 min and then used for LC-MS measurement.

#### 2.2.2 Lipidome extraction

The samples were stored in 1.5 mL of PBS at -80°C. To prepare the sample for analysis, 50 μL of sample was added into 300 μL of methanol, and the mixture was vortexed for 120 s. Then, 900 μL of methyl tert-butyl ether (MTBE) and 250 μL of ultrapure water were added to the mixture, which was shaken for 15 min at room temperature and incubated at 4°C for 30 min until separation. For LC-MS detection, 900 μL of the upper liquid layer was removed and dissolved in 600 μL of acetonitrile isopropanol mixture.

### 2.3 LC-MS/MS analysis

#### 2.3.1 Untargeted metabolomics analysis

Because of the wide variety of metabolites, untargeted metabolomics analysis was performed using two analytical methods in order to obtain more metabolites. For method 1, the samples were determined on TSQ Quantiva (Thermo, CA) using C18-based reversed-phase chromatography with mobile phase A (10 mM tributylamine and 15 mM acetic acid in water) and mobile phase B (100% methanol). Each sample was eluted in a gradient from 5% to 90% using mobile phase B for 25 min. The resolution was 0.7 full width at half maximum (FWHM) for both Q1 and Q3. The ion source voltage was 3500 V for positive ion mode (ESI+) and 2500 V for negative ion mode (ESI-). The ion transfer tube temperature was set to 350°C, and the heater temperature was 300°C. The sheath gas flow rate was set to 35 arb, and the auxiliary gas flow rate was set to 10 arb.

For method 2, Liquid Chromatography Ultimate 3000 UHPLC (Dionex) with Q Exactive quadrupole-orbitrap high-resolution mass spectrometer system (Thermo Scientific, USA) was utilized. In ESI+ mode (voltage 3.5 kV), Atlantis HILIC silica column (2.1 × 100 mm, Waters Corporation) was used with mobile phase A (10 mM ammonium formate, 50 mL of ultrapure water, 950 mL of HPLC grade acetonitrile, and 1 μL of formic acid) and mobile phase B (10 mM ammonium formate, 500 mL of ultrapure water, 500 mL of HPLC grade acetonitrile, and 1 μL formic acid) for separation. The linear gradient of the mobile phase was as follows: 0 min, 1% B; 2 min, 1% B; 3.5 min, 20% B; 17 min, 80% B; 17.5 min, 99% B; and 19 min, 99% B. Moreover, 70,000 FWHM and 17,500 FWHM were used for full-scan and MS/MS data acquisition, respectively. The column temperature, the sheath gas flow rate, and the auxiliary gas flow rate were set to 275°C, 35 arb, and 10 arb, respectively (18).

#### 2.3.2 Untargeted lipidomics analysis

The sample data were acquired on Ultimate 3000 UHPLC (Dionex) with QExactive Orbitrap mass spectrometer (Thermo, CA) in tandem with Cortecs C18 column (1.6 Lm, 2.1 × 50 mm; Waters Corporation). The mixture of ultrapure water containing 10 mM ammonium acetate and chromatographic acetonitrile (v/v=4:6) was used as mobile phase A. The mixture of chromatographic acetonitrile and isopropanol (v/v=1:9) was used as mobile phase B. The voltages of ESI+ mode ion source and ESI- mode ion source were 3.2 kV and 2.8 kV, respectively. Lipids were structurally characterized by acquiring data-dependent MS2 spectra with the following key settings: 70,000 FWHM; 17,500 MS/MS resolution; cycle counting of 10; 240-2000 scan mass range (m/z) for ESI+ mode; and 200-32000 scan mass range (m/z) for ESI- mode (18).

### 2.4 Data processing and statistical analysis

#### 2.4.1 Data processing

Polar metabolites were identified simultaneously by Tracefinder at two levels as follows: 1) by accurate molecular weight identification of the metabolites; and 2) by comparison of the fragments with a secondary mass spectrometry database. The resulting metabolites were identified after primary and secondary mass spectrometry, and the mass deviations of parent and self ions were set to 10 ppm and 15 ppm, respectively. A drift of 0.25 min retention time was allowed during the quantification.

Lipid metabolites were identified qualitatively by Lipidsearch software (Thermo Scientific, USA), and fragment information was compared to the platform’s own database for various phospholipids, neutral glycerolipids, sphingolipids, neutral glycosphingolipids, glycosphingolipids, sterols, and fatty esters. The mass deviations of the parent ion and the characteristic fragment were set at 5 ppm and 10 ppm, respectively. Only peaks with peak areas greater than 5E6 were identified as valid identifications. A retention time drift of 0.25 min was allowed during the quantification.

#### 2.4.2 Statistical analysis

The metabolomics and lipidomics data were normalized separately before further processing. The quantitative information obtained by the above methods was then integrated, and duplicate data were eliminated to ensure uniqueness of metabolites and lipids. After removing the duplicated substances, 309 metabolites and 441 lipidosomes were obtained from metabolomics and lipidomics, respectively. Multivariate analysis, including principal component analysis (PCA), OrthoPLS-DA (OPLS-DA) and univariate analysis, including independent samples t-test and fold change, were performed on the MetaboAnalyst website (https://www.metaboanalyst.ca/). The FDR (Benjamini-Hochberg) adjusted p.value < 0.05 were considered significant.

### 2.5 RNA extraction and quantitative real-time PCR

Total RNA was extracted from testicular tissue (10 mg) with TRIzol reagent (Takara, Tokyo, Japan) according to the manufacturer’s instructions. A Prime-Script RT Reagent Kit (Takara, Japan) was used to reverse transcribe RNA into cDNA, and SYBR Premix Ex Taq (Takara, Japan) was used to perform qRT-PCR utilizing a Thermal Cycler Dice Real-Time System (Takara, Japan). The following thermocycler program was used: 95°C for 5 min; 40 cycles of 95°C for 10 s, 60°C for 10 s, and 72°C for 10 s; melting curve from 95 to 60°C; and 37°C for 10 s. β-actin was used as an endogenous control to normalize the data, and the 2^-ΔΔCt^ calculation method was employed to analyze the data. The qRT-PCR primers are listed in **Supplemental Table 1**.

### 2.6 Western blot analysis

For total cellular protein extraction, cells were lysed with ice-cold radioimmunoprecipitation assay (RIPA) buffer supplemented with protease and phosphatase inhibitors (Shenergy Biocolor Bioscience & Technology Company, Shanghai, China). Equal amounts of proteins were loaded on 12% sodium dodecyl sulfate-polyacrylamide gel electrophoresis (SDS-PAGE) and transferred to a nitrocellulose membranes (Millipore, Billerica, MA, USA). The membranes were blocked by PBS supplemented with 0.1% Tween 20 and 5% non-fat dry milk (PBST-milk) for 1 h at room temperature. The membranes were then incubated with primary antibodies against Cleaved-Caspase 3, Bax, and Bcl-2 (antibodies listed in **Supplemental Table 2**) at 4°C overnight. The membranes were then washed with TBS containing Tween-20 (TBST) followed by incubation of anti-rabbit or anti-mouse IgG secondary antibody conjugated to horseradish peroxidase (1:5000) for 1 h at room temperature. The protein bands were visualized by a HyGLO HRP detection kit (Denville, NJ, USA), and protein expression levels were quantified using Fluor Chem Q SA software. Tubulin was used as a loading control.

### 2.7 Flow cytometry analysis of apoptosis

For apoptosis analysis, cells were trypsinized, washed with cold PBS, and resuspended in fluorescence-activated cell-sorting (FACS) buffer containing 2% FBS. Cells were then treated with the FITC-Annexin V Apoptosis Detection kit with 7-AAD (BioLegend, Inc.) for 30 min at 4°C. Apoptosis (7-AAD) was analyzed using flow cytometry and Cell Quest software (version 5.1; BD Biosciences).

## 3 Results

### 3.1 Global profiles of metabolism in SCs after PA treatment

**Figure 1:**
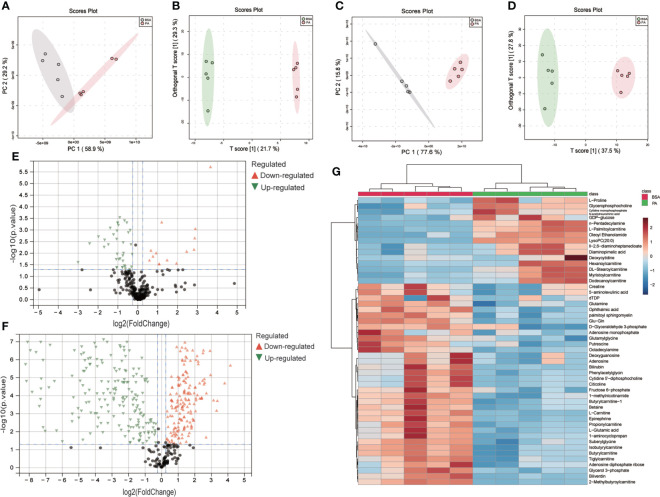
Metabolomics and lipidomics revealed global changes to metabolites in PA-treated SCs. PCA and OPLS-DA score plots between the BSA group and PA group in metabolomics **(A, B)** and lipidomics **(C, D)**. Volcano plots of the BSA group vs. the PA group in metabolomics **(E)** and lipidomics **(F)**. Heatmap showing differential polar metabolites in SCs treated with PA **(G)**.

To study the global profiles of metabolism in SCs treated by PA, we conducted a multivariate analysis at first. A clear separation was observed in PCA and OPLS-DA models between the PA and BSA groups (**Figures 1A–D**), indicating distinct changes between the BSA group and PA group. Then we performed the univariate analysis of these data, identified 50 polar metabolites and 349 lipid metabolites as differential, according to the cutoffs of p.adj < 0.05 and fold change > 1.2. After PA treatment, 14 polar metabolites and 204 lipid metabolites were upregulated in SCs, whereas 36 metabolites and 145 lipid metabolites were downregulated in SCs (**Figures 1E, F**). Among the differential polar metabolites, there were metabolites associated with energy metabolism, such as the TCA cycle and fatty acid β-oxidation, prompting our further investigation of the effect of PA on energy metabolism in SCs (**Figure 1G**).

### 3.2 PA overload does not affect glucose metabolism in SCs

**Figure 2:**
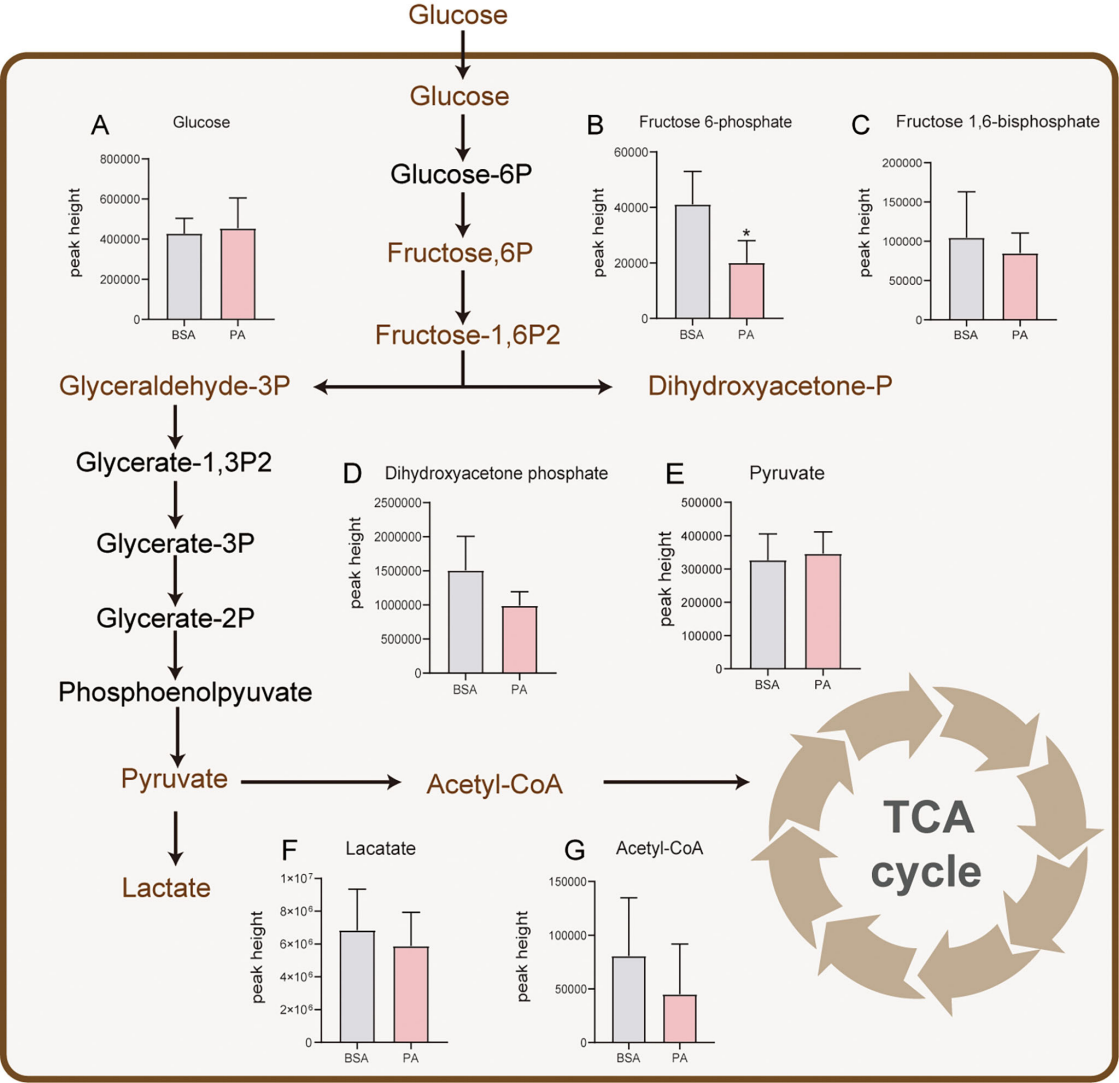
Effect of PA on glucose and lactate metabolism of SCs. After glucose is taken up by SCs, pyruvate is produced through glycolysis. Most of the pyruvate is used to produce and secrete lactate for GCs to provide energy, and only a small part of pyruvate participates in the TCA cycle. Levels of glucose **(A)**, fructose-6-phosphate **(B)**, fructose-1,6-bisphosphate **(C)**, dihydroxyacetone phosphate **(D)**, pyruvate **(E)**, lactate **(F)**, and acetyl-CoA **(G)**. The metabolites marked in brown were detected by metabolomics analysis. The red columns represent the PA group, and the grey columns represent the BSA group. Data were expressed as mean ± SD, *p < 0.05.

SCs produce the preferred energy substrate for spermatocytes and spermatids through glucose metabolism (19). To explore whether PA affects the glucose metabolism pathway in SCs, we analyzed the expression of related metabolites in glucose metabolism. Seven metabolites, namely, glucose, fructose-6-phosphate, fructose-1,6-bisphosphate, dihydroxy-acetonephosphate, pyruvate, lactate, and acetyl-CoA, related to glucose metabolism were detected. There was little difference between the PA group and BSA group, except for fructose-6-phosphate, which was decreased after PA treatment (**Figures 2A–G**). These findings suggested that PA may not cause significant changes in the glucose metabolic pathway of SCs.

### 3.3 PA overload promotes the accumulation of long-chain acylcarnitines in SCs

**Figure 3:**
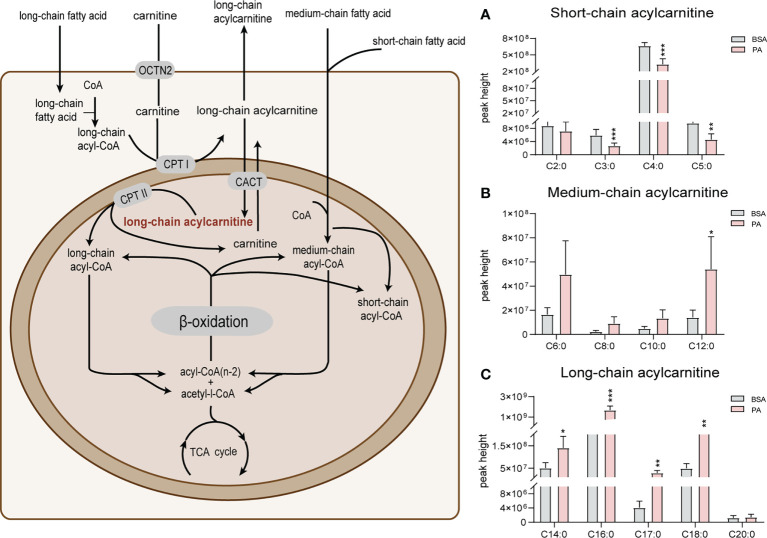
Effect of PA on acylcarnitines in SCs. The schematic diagram of fatty acid β-oxidation in SCs is shown on the left. Free carnitine in plasma enters cells through organic cation/carnitine transporter 2 (OCTN2). Long-chain fatty acids then enter the mitochondria with the help of carnitine and are transformed into acylcarnitines by carnitine palmitoyltransferase 1 (CPT1), which is located in the outer membrane of mitochondria. Acylcarnitines then pass through the inner mitochondrial membrane (IMM) through carnitine acyltransferase (CACT) to exchange free carnitine. Carnitine palmitoyltransferases 2 (CPT 2), which is located in the IMM, converts acylcarnitines into acyl-CoA and free L-carnitine for the next oxidation reaction. Medium- and short-chain fatty acids do not require carnitine to enter mitochondria for oxidation. Subsequently, acylcarnitines are oxidized in the form of acyl coenzyme A (acyl-CoA) by a series of enzymes to produce acyl-CoA with two less carbon atoms than the original and acetyl-CoA, which is finally oxidized by the TCA cycle to produce CO_2_ and H₂O as well as to release energy. The changes in long-, medium- and short-chain acylcarnitines are shown in panels **A–C**, respectively. Data were expressed as mean ± SD, *p < 0.05, **p < 0.01, ***p < 0.001.

Fatty acid β-oxidation is considered as the main energy source of SCs (20). Metabolomics data indicated that several long-chain acylcarnitines (C14:0, C16:0, C17:0, and C18:0) significantly accumulated after PA stimulation (**Figure 3A**). In addition, the level of medium-chain acylcarnitines (C6:0, C8:0, C10:0, and C12:0) increased after PA treatment (**Figure 3B**). Compared to medium- and long-chain acylcarnitines, short-chain acylcarnitines (C2:0, C3:0, C4:0, and C5:0) were notably decreased in SCs exposed to PA (**Figure 3C**). The aggregation of long-chain acylcarnitines indicated the fatty acid β-oxidation in SCs was suppressed after PA treatment.

### 3.4 PA overload does not result in amino acid metabolism disorder in SCs

**Figure 4:**
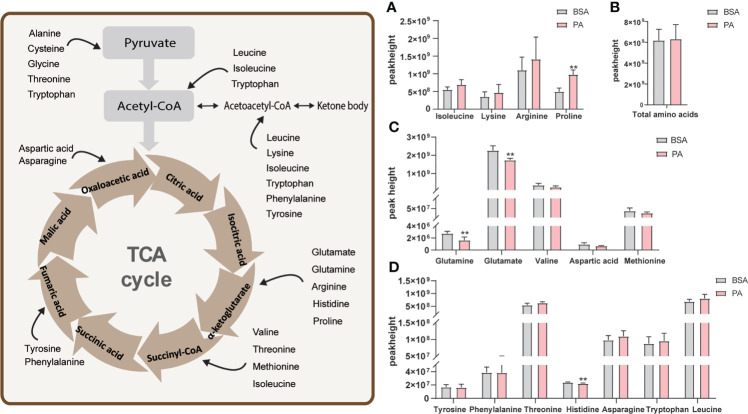
Effect of PA on amino acid metabolism of SCs. The schematic diagram of amino acids participating in the TCA cycle is shown on the left. Amino acids enter the TCA cycle through conversion into different substances. Peak height of amino acids that participate in the TCA cycle **(A, C, D)** and the total amount of these amino acids **(B)**. Data were expressed as mean ± SD, **p < 0.01.

Some amino acids supply energy to SCs *in vivo* by participating in TCA cycle (9). Metabolomics analysis identified 16 amino acids that participated in the TCA cycle in SCs. Using FC values > 1.2 or < 0.83 as criteria, these amino acids were divided into three groups (**Figures 4A, C, D**). As shown in **Figure 4A**, isoleucine, lysine, proline, and arginine were detected, but the p.adj was > 0.05 for these amino acids, except for proline. **Figure 4C** shows that there were five amino acids with FC values < 0.83, but only glutamate and glutamine were significantly declined in PA-treated cells. Compared to the BSA group, the levels of histidine, tyrosine, phenylalanine, and tryptophan did not change in the PA group (0.83 < FC < 1.2) (**Figure 4D**). Furthermore, the total amount of these amino acids did not change in the PA group (**Figure 4B**).

### 3.5 PA overload leads to a significant change in lipid compositions

**Figure 5:**
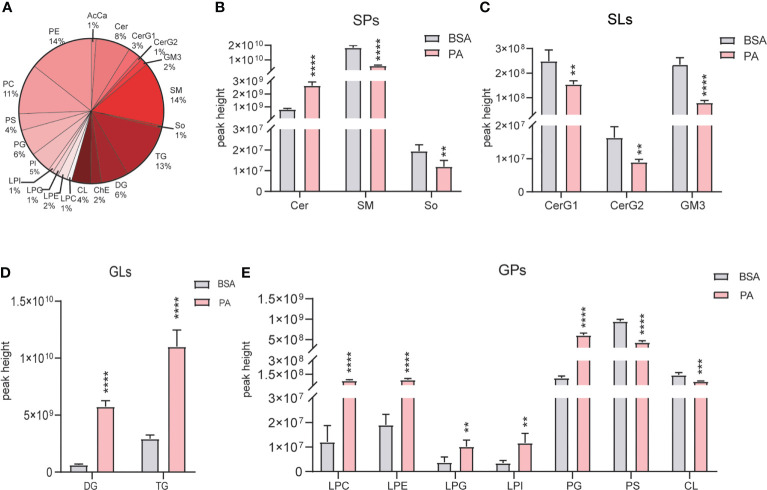
Overall lipid profiles of SCs treated with PA and BSA. **(A)** Percentage of lipid subclasses detected in SCs. The peak height of GLs **(B)**, SPs **(C)**, GLs **(D)**, and GPs **(E)** in the PA group and BSA group. Data were expressed as mean ± SD, **p < 0.01, ***p < 0.001, ****p < 0.0001.

Lipidomics analysis detected 20 lipids (**Figure 5A**), and they were divided into the following 4 categories: sphingolipids (SPs), saccharolipids (SLs), glycerolipids (GLs), and glycerophos-pholipids (GPs). Regarding SPs, ceramide (CER) was significantly increased, while sphingomyelin (SM) and sphingosine (So) were reduced (**Figure 5B**). Moreover, glucosylceramide (CerG1), diglycosylceramide (CerG2), and ganglioside (GM3) were decreased in SLs (**Figure 5C**). The levels of both diglyceride (DG) and triglyceride (TG) were accumulated in GLs (**Figure 5D**). Regarding GPs, Lyso-PC (LPC), Lyso-PE (LPE), Lyso-PG (LPG), Lyso-PI (LPI), phosphatidylcholine (PC), and phosphatidylglycerol (PG) were elevtaed, while cardiolipin (CL) and phosphatidylethanolamine (PE) were notably decreased (**Figure 5E**).

### 3.6 PA overload promotes SCs apoptosis

**Figure 6:**
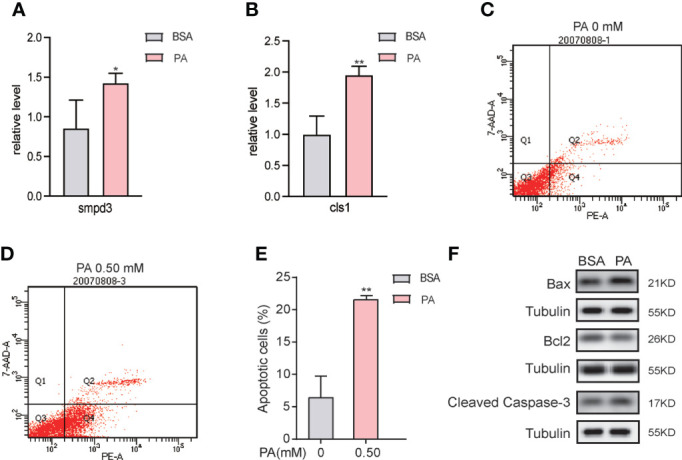
PA overload promotes apoptosis in SCs. **(A, B)** mRNA expression levels of smpd3 and cls1 in PA-treated SCs. **(C–E)** After PA treatment at concentrations of 0 mM and 0.5 mM for 24 h, the apoptosis rate of SCs was detected by flow cytometry (FCM). **(F)** Apoptosis-related protein expression in PA-treated SCs. 7-AAD, 7-aminoactinomyosin D. Data were expressed as mean ± SD, *p < 0.05, **p < 0.01.

The combined metabolomics and lipidomics data suggested that apoptosis was triggered in PA-treated SCs. We first validated the lipidomics results and found that the mRNA level of smpd3, which encodes neutral sphingomyelinase (nSMase), was elevated (**Figure 6A**). However, the mRNA level of cls1, a gene encoding CL synthase, was enhanced (**Figure 6B**). Next, we evaluated SCs apoptosis after PA treatment. As presented in the **Figures 6C, D, E**, PA treatment significantly increased the apoptosis rate of SCs. We further identified the expression of Cleaved Caspase-3, Bcl-2, and Bax by western blot analysis. Compared to the BSA group, the protein expression levels of Bax and Cleaved Caspase-3 were significantly upregulated in the PA group, while the expression level of Bcl-2 was downregulated (**Figure 6F**), which indicated that PA induced apoptosis of SCs.

## 4 Discussion

The negative effects of lipotoxicity on male fertility has gained attention in recent years. As is well known, SCs are an indispensable part in male reproduction (14). However, relevant studies focusing on the influence of lipotoxicity on energy metabolism in SCs are limited. Here, we treated SCs with PA to comprehensively analyze the effect of lipotoxicity on the energy metabolism of SCs through metabolomics and lipidomics analysis. Because the proportion of SCs in testes is low (only 3–5%), a whole testis is not sufficient to conduct analysis to reflect the changes in SCs. Thus, we extracted primary SCs for more detailed study and analysis. The present study demonstrated that PA inhibits mitochondrial function and triggers apoptosis by inhibiting fatty acid β-oxidation in SCs.

Metabolomics is a method that accurately analyzes the dynamic metabolic changes in cells, tissues, and whole organisms. We performed metabolomics analysis on the related substances of the three major energy metabolites in cells. In the process of spermatogenesis, SCs only use a small part of glucose to meet their own energy needs but convert most of the glucose into lactate, which is used for the GC energy supply (21). In the present study, we found that most of the metabolome features detected for the glucose metabolism pathway, such as pyruvate and lactate, were not significantly changed in PA-treated SCs. Both our previous and present study found that the intracellular lactate level was not changed (12), suggesting that the glucose metabolism of SCs is not affected by PA and that they still maintain the normal supportive function for GCs.

Fatty acids are the main energy substrate of SCs (22). It has been reported that 66% of PA is processed by fatty acid β-oxidation to generate CO_2_ in SCs (20). The accumulation of long-chain fatty acylcarnitines has been regarded as a manifestation of the inhibition of fatty acid β-oxidation (23). The present study showed that a large amount of long-chain fatty acylcarnitines accumulated after PA treatment, indicating that fatty acid β-oxidation in SCs is hindered by PA. We have previously reported that PA increases the expression of carnitine palmitoyltransferase 1α (CPT1α) and long chain acyl-CoA dehydrogenase (LCAD) at the mRNA and protein levels (12). Combined with the present results, we propose that after PA stimulation, SCs become active in the β-oxidation of fatty acids due to the increased uptake of fatty acids. However, excess PA impairs mitochondrial function and eventually inhibits the β-oxidation of fatty acids, causing sustained accumulation of medium- and long-chain acylcarnitines as well as a decrease in short-chain acylcarnitines.

The major functions of amino acids in SCs include generating protein, participating in the TCA cycle to generate energy, and participating in lipid production (9). Some studies have confirmed that valine, leucine, alanine, glutamine, and glutamate can be used as independent substrates to provide energy for SCs to maintain high-speed metabolic activities (9), (24). Metabolomics analysis indicated that the levels of glutamine and glutamate were significantly decreased. However, we were unable to determine if the PA-induced reduced amino acids were involved in the TCA cycle, indicating that further investigation is required. Interestingly, the total amount of amino acids that participated in the TCA cycle did not change, suggesting that PA treatment may not affect the amino acid metabolism in SCs and that amino acid metabolism does not compensate for the deficient energy supply due to fatty acid oxidation.

Because the energy supply was influenced, we speculated that the function of SCs was affected. To further explore the effect of PA on SCs, lipidomics analysis was performed on SCs. Accumulation of FFA in the TG pool is thought to be a buffering effect against lipotoxicity (25). In the present study, lipidomics analysis demonstrated that the levels of TG and DG in SCs were increased after PA treatment, illustrating that SCs may counteract the harmful effects of lipotoxicity for a short period of time. However, after the protective effect disappears, intracellular fatty acids will continue to exert their destructive effect on the cells. In addition, large amounts of PA inhibit TG synthesis at the DG stage, allowing DG to accumulate in cells (26). The present findings suggested that the protective effect of SCs is ceased and that lipotoxicity begins to have a progressive and devastating effect.

High concentrations of PA lead to upregulation and increased activity of nSMase and serine palmitoyl transferase (SPT), resulting in increased *de novo* synthesis of CER and release of So from the cell membrane to convert to CER (22). It has been reported that the addition of CER to the medium inhibits the mitochondrial respiratory chain and induces voltage-dependent anion channel closure, which leads to mitochondrial dysfunction (20), altered expression of Bcl-2 family proteins, and ROS generation, eventually resulting in apoptosis (27, 28). In addition, phospholipids can be metabolized from PA and continue to be metabolized to lysophospholipids, which promote apoptosis through various pathways (27, 29, 30). Moreover, another component of GLs, CL, is contained in the inner mitochondrial membrane (IMM), and it is not found in any other organelle. In healthy mitochondria, CL interacts with proteins to support cristae, stabilize respiratory chain complexes, and regulate autophagy in the IMM. Hence, decreased CL is a marker of impaired mitochondrial function (31, 32).

The present study demonstrated that the production of CER and lysophospholipids was increased in SCs after PA treatment, while the CL content decreased significantly. In addition, the mRNA level of smpd3, which encodes nSMase, was elevated, thereby verifying that the synthesis of CER increases after PA stimulation. The increased mRNA level of cls1, a gene encoding CL synthase, may indicate a compensatory upregulation of CL remodeling and mitochondrial biogenesis in SCs treated by PA. Furthermore, it is unknown whether CL lipid precursors are downregulated in PA-treated SCs, resulting in a decrease in total CL despite the upregulation of cls1 mRNA (33). We have previously stained SCs with MitoSOX and MitoTracker, demonstrating that there is increased mitochondrial ROS levels in PA-treated SCs (12). In addition, we have previously used an extracellular flux analyzer to demonstrate that the ATP level is significantly reduced and that the proton leak is significantly increased in SCs treated with PA (12). Thus, the present study further confirmed our hypothesis of impaired mitochondrial function induced by PA.

Mitochondria play a key role in cell death and its regulation, and Bcl-2 family proteins control mitochondrial outer membrane permeability by regulating membrane potential (34). The imbalance of Bcl-2 family expression results in the release of proapoptotic factors into the cytoplasm, thereby inducing apoptosis (35). The present study demonstrated that PA treatment of SCs reduced cell viability, upregulated Bax and Cleaved Caspase-3 expression, and downregulated Bcl-2 expression. These results indicated that PA induces apoptosis through the imbalance of Bcl-2 family proteins in SCs.

In summary, we conducted an in-depth study of changes in the energy metabolism of SCs under the effect of PA using a combination of metabolomics and lipidomics analyses. The results showed that fatty acid β-oxidation of SCs is disrupted after PA treatment. Although SCs are still able to provide energy for GCs under PA stimulation, their own source of energy supply is inhibited, causing mitochondrial damage, leading to apoptosis.

## Data availability statement

The original contributions presented in the study are included in the article/**Supplementary Material**, further inquiries can be directed to the corresponding author/s.

## Ethics statement

The animal study was reviewed and approved by Animal Ethics Committee of Shandong Provincial Hospital.

## Author contributions

XX was responsible for the completion of molecular biology experiments and the draft of the article. DL completed the collection and arrangement of experimental data. QX and PL completed the production of the chart. CY was responsible for the design, guidance of the experimental techniques and manuscript correction. QG completed the overall thinking and supervision of the project. All authors contributed to the article and approved the submitted version.

## Funding

This work was supported by grants from the National Natural Science Foundation (81770860 and 81641030) and the Key Research and Development Plan of Shandong Province (2017CXGC1214).

## Conflict of interest

The authors declare that the research was conducted in the absence of any commercial or financial relationships that could be construed as a potential conflict of interest.

## Publisher’s note

All claims expressed in this article are solely those of the authors and do not necessarily represent those of their affiliated organizations, or those of the publisher, the editors and the reviewers. Any product that may be evaluated in this article, or claim that may be made by its manufacturer, is not guaranteed or endorsed by the publisher.
