# Supplementary material for: Atlas of metabolism reveals palmitic acid results in mitochondrial dysfunction and cell apoptosis by inhibiting fatty acid β-oxidation in Sertoli cells
Source: Front Endocrinol (Lausanne). 2022 Sep 27;13:1021263. doi: 10.3389/fendo.2022.1021263 (PMC9552013; doi:10.3389/fendo.2022.1021263)
Supplement: Supplementary file 1 [file DataSheet_1.docx]

Supplementary Material

# Supplementary Figures and Tables

## Supplementary Figures


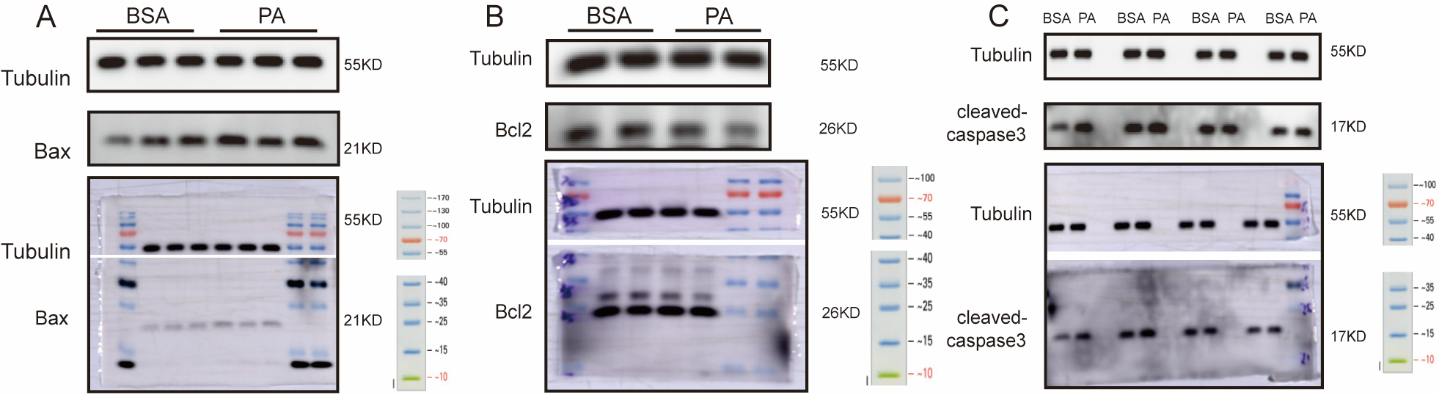


**Supplementary Figure1** The cropped images of blots are shown in figure 5F. And the full scan of the entire original gel(s) are shown in A, B, C above.

## Supplementary Tables

**Supplementary Table1**. List of Primers Used for qRT-PCR

| **Gene** | **Forward Primer** | **Reverse Primer** |
| --- | --- | --- |
| **Smpd3** | **ACACGACCCCCTTTCCTAATA** | **GGCGCTTCTCATAGGTGGTG** |
| **Cls** | **TGGATGGATTTATTGCTCGAAA** | **TGGGACTGGAATAAGATCTGCAT** |
| **β- actin** | **CTAAGGCCAACCGTGAAAAGA** | **CCAGAGGCATACAGGGACAAC** |

**Supplementary Table2**. List of Antibodies Used for Western blotting

| **Antibodies/Reagent** | **Source** | **Identifier** |
| --- | --- | --- |
| **cleaved-caspase3** | **Cell Signaling Technology** | **9664** |
| **Bax** | **Proteintech** | **50599-2-AP** |
| **Bcl2** | **Proteintech** | **26593-1-AP** |
| **Tubulin** | **Proteintech** | **66031-1-Ig** |
